# Barriers and facilitators to HIV index contact testing in facility and community settings

**DOI:** 10.4102/sajhivmed.v26i1.1733

**Published:** 2025-10-31

**Authors:** Nyeleti Pretty Chauke, Cathrine Chinyandura, Fezile Buthelezi, Anele Jiyani, Kate Rees

**Affiliations:** 1 Anova Health Institute, Johannesburg, South Africa; 2 Department of Community Health, School of Public Health, Faculty of Health Sciences, University of the Witwatersrand, Johannesburg, South Africa

**Keywords:** index contact testing, implementation barriers, implementation facilitators, HIV testing, community testing, status neutral testing

## Abstract

**Background:**

The WHO emphasises contact investigation of people with HIV for case finding. However, satisfactory implementation of index contact testing remains challenging in Johannesburg.

**Objectives:**

This study draws from the Consolidated Framework for Implementation Research to explore healthcare workers’ (HCWs) insights on barriers and enablers of index contact testing implementation.

**Method:**

Data were collected through semi-structured interviews, at four healthcare facilities and four non-profit organisations (NPOs) in Johannesburg, from October 2023 to November 2023, and analysed thematically, guided by the Consolidated Framework for Implementation Research framework.

**Results:**

Twenty-three HCWs participated: 18 counsellors (78.3%) and 5 nurses (21.7%). Most participants were aged 20–40 years (56.5%). Participants were drawn from facilities (65.0%) and NPOs (35.0%). Counsellors demonstrated a more nuanced understanding of index contact testing. Major barriers were identified in the innovation, outer setting, and inner setting domains, and key enablers in the innovation and process domains. Innovation domain barriers included perceived complexity of guidelines and their time-consuming nature. Key enablers were adaptations to educate clients about pre-exposure prophylaxis (PrEP), incorporating index contact testing into pre-test counselling, and offering home-based testing for contacts. Outer setting barriers included fears of intimate partner violence (IPV), stigma, and geographic dispersion of contacts. Inner setting barriers were limited training, insufficient knowledge, and inadequate HCW skills, compounded by a lack of resources.

**Conclusion:**

This study highlights critical barriers that need to be addressed, through simplified guidelines, targeted training, resource allocation, stigma reduction, and IPV-reduction strategies. Leveraging enablers like PrEP education and home-based testing can enhance implementation and engagement.

**What this study adds:** HCWs experienced barriers to index contact testing implementation, despite scale-up. We recommend simplifying guidelines, providing ongoing, targeted training, including on managing IPV and stigma, and leveraging interest in status-neutral approaches like offering PrEP.

## Introduction

Index contact testing, one of the solutions proposed to achieve the first UNAIDS 95-95-95 target,^1^ is a case-finding approach that identifies people who are vulnerable to HIV infection, such as sexual or needle-sharing partners and biological children of people living with HIV (PLHIV), and offers them HIV testing.^2,3,4^ The WHO recommends contact investigation of index individuals as a key approach to enhance HIV case detection.^5^ Index contact testing allows for the early detection of HIV in individuals who may have been exposed to the virus, helps break the chain of transmission, and enables targeted outreach efforts toward specific populations at higher risk, such as people in relationships with mixed HIV status and children of PLHIV.

Index contact testing is effective in multiple settings, including healthcare facilities and in communities.^6,7,8,9,10^ There are several ways in which contacts can be linked to HIV testing during index contact testing. Broadly, passive referral, which is also known as ‘client referral’, is where the index client independently facilitates their contact’s testing, and provider referral is where healthcare workers (HCWs) actively reach out to the index client’s contacts.

Despite the rollout of index contact testing by the National Department of Health in South Africa, in Johannesburg, index contact testing contributed about 9.5% of case finding in 2023 (Programme data, 27 November 2024), falling well short of the 40% target. The implementation of index contact testing is influenced by various client-related, HCW-related, and facility-related factors. On the client side, barriers include reluctance to disclose partner information or to undergo testing because of the fear of relationship breakdown or societal stigma, high mobility from activities such as cross-border trade or farming, and incomplete or inaccurate contact details. Adolescent clients and key populations often face challenges in providing information on casual partners, further complicating tracing efforts.^1,11^ Among HCWs, inadequate training in index contact testing strategies, limited elicitation skills, and insufficient knowledge hinder effective implementation. Additionally, heavy workloads limit the time available for thorough index contact elicitation. However, HCWs with strong interpersonal skills, non-stigmatising attitudes, and a sense of purpose enhance the feasibility of index contact testing.^11,12^ Facility-related challenges include privacy concerns because of a lack of private spaces, resource limitations such as insufficient transport for contact tracing, and logistical constraints in tracing contacts.^1,11^

In Johannesburg, fewer than half of elicited index contacts undergo testing in both facility and community settings (Programme data, 20 July 2023). Therefore, understanding the barriers and facilitators to implementation is crucial for developing context-specific strategies and innovative solutions. Addressing these issues can optimise underutilised resources, strengthen index contact testing initiatives, and ultimately improve programmatic outcomes. This study aimed to explore barriers and facilitators to index contact testing in Johannesburg, including acceptability of recommended referral approaches.

## Research methods and design

### Study design and setting

This study employed a qualitative design with a deductive approach, supplemented with routine data. A qualitative approach includes in-depth exploration, allowing for a ‘deep delve’ into participants’ views.^13,14,15^ This approach aimed to gain contextual understanding by exploring the factors affecting index contact testing.

The study was conducted in the South of Johannesburg, sub-district G, in four healthcare facilities and four non-profit organisations (NPOs). Sub-district G forms the extreme southern boundary of Greater Johannesburg along its eastern, western, and southern edges. Located approximately 40 kilometres south of the city centre, it is the most isolated and least integrated region within Johannesburg.^16,17^ The region is marked by extensive open spaces, uncultivated farmland, agricultural holdings, and undeveloped land.^17^ The City of Johannesburg faces social and structural factors such as poverty, unemployment, limited education, and gender inequality that heighten vulnerability to HIV and tuberculosis while hindering prevention and treatment efforts. These challenges, particularly for those with lower socio-economic status, affect health-seeking behaviour and adherence to treatment.^18^ There were an estimated 623 200 PLHIV in Johannesburg in 2023.^19^

### Conceptual framework

The framework employed in this study is the updated Consolidated Framework for Implementation Research by Damschroder et al.,^20^ which aims to identify and explain the barriers and facilitators that influence the effectiveness of implementation. Implementation research frameworks, such as Consolidated Framework for Implementation Research, are essential and widely employed in guiding assessments of contextual determinants of implementation.^20,21^ The Consolidated Framework for Implementation Research consists of five domains: the innovation, outer setting, inner setting, individual, and implementation process:

1. The innovation domain focuses on the specific initiative or intervention being introduced; in this case, it refers to the index contact testing modality.
2. The outer setting encompasses the broader external context impacting the innovation, such as community, system, or state-level factors influencing implementation success. In this study, the outer setting refers to external factors beyond healthcare facilities and NPOs, such as societal attitudes.
3. The inner setting describes the immediate environment where the innovation is applied, such as a healthcare facility or NPO, and includes factors within these organisations.
4. The individual domain focuses on the roles and characteristics of the individuals involved, highlighting the personal attributes, attitudes, and behaviours of HCWs who implement index contact testing.
5. The implementation process refers to the activities and strategies employed to introduce and sustain index contact testing, including specific steps and actions that facilitate its integration.

Methodologically, this study was rooted in phenomenology, which seeks to understand participants’ lived experiences and perspectives.^22,23^ This orientation was particularly relevant given the focus on HCWs’ personal experiences with implementing index contact testing, rather than on broader cultural or ethnographic accounts. By combining phenomenology with the principles of Implementation Science and the Consolidated Framework for Implementation Research, this study not only explored individual experiences but also situated these within the organisational and systemic contexts that influence implementation outcomes.

### Study population and sampling

Four facilities and four NPOs in Johannesburg sub-district G were purposively selected as the study sites, with the inclusion of all NPOs operating in the sub-district. Eighteen lay counsellors and five nurses were purposively recruited, based on their direct involvement in the implementation of index contact testing. The sample size was estimated based on past experience of data saturation in this setting.^24,25^ We determined that saturation had been reached when ideas on barriers and facilitators recurred and new interviews did not generate additional information. This was determined by the data collectors in collaboration with the rest of the study team. Among the four purposefully selected healthcare facilities, two were classified as high performing, while the other two were identified as underperforming. High-performing facilities were those that met programmatic targets for index contact testing, such as achieving a high index elicitation rate (≥ 80% of eligible index clients identified and offered index contact testing), high contact testing uptake (≥ 80% of elicited contacts tested), and high HIV yield (≥ 5% – 10% positivity rate among contacts tested), along with timely linkage to care for those testing positive. Underperforming facilities were those that fell substantially below these targets, with lower index elicitation (< 80%), poor contact testing uptake (< 80%), low HIV yield (< 5%), and/or delays or gaps in linkage to care.

### Data collection

Data were collected through individual semi-structured in-person interviews at participants’ workplaces (facilities and NPOs) between October 2023 and November 2023 by N.P.C., A.J., and F.B. The interview guide included how participants conduct index contact testing, methods used to solicit contacts, strategies for encouraging index clients to bring in their contacts, preferred modes of contact referral, attendance at index training, adherence to index guidelines, suggested improvements for enhancing index contact testing, expectations regarding support from leaders, current support received, the influence of beliefs and values on index contact testing implementation, and challenges faced in eliciting index contacts. The interviews lasted between 30 min and 60 min, were conducted in person in isiZulu, SeSotho, and/or English, and were audio recorded with participants’ permission. N.P.C. and F.B. were fluent in all three languages, while A.J. was fluent in isiZulu and English. Accordingly, A.J. conducted interviews in isiZulu and/or English, whereas N.P.C. and F.B. conducted interviews in isiZulu, SeSotho, and/or English. Following the interviews, the recordings were translated into English, where applicable, and transcribed by the three researchers.

### Data analysis

Data were thematically analysed using the framework established by Braun and Clarke^26^ and Braun, Clarke and Rance,^27^ following a deductive approach,^28^ with the Consolidated Framework for Implementation Research domains serving as predetermined themes. The transcripts were coded and analysed using Taguette software^29^ by the researchers (N.P.C., A.J., and F.B.), with identified sub-themes categorised according to the five domains and corresponding constructs of the Consolidated Framework for Implementation Research framework. Coding involved creating codes to represent concepts to be tracked. Relevant text passages in the documents were highlighted and assigned the appropriate codes, with multiple codes applied to a single passage where necessary. After coding, the work was reviewed and refined by the analysis team. Finally, the coded data was exported to Excel for further analysis, facilitating a deeper exploration of the themes within the data. This approach facilitated a comprehensive understanding of the barriers and facilitators affecting the implementation of index contact testing. Intercoder reliability was achieved by developing a clear coding framework, ensuring all researchers applied consistent codes, and comparing their results. Discrepancies were resolved through multiple discussions; regular meetings ensured consistency in coding.

### Researcher characteristics and reflexivity

The research team comprised three researchers (two female and one male) trained in qualitative methods, with professional backgrounds in HIV testing services and public health. N.P.C. had prior experience working in healthcare settings and with HIV testing programmes, which enhanced contextual understanding but also introduced potential biases. A.J. and F.B. had experience as researchers and conducting health-related research in healthcare facilities with HCWs. To mitigate this, reflexivity was prioritised throughout the study: the researchers acknowledged their dual roles as professionals and investigators, engaged in ongoing self-reflection, and discussed how their assumptions and professional experiences might influence data collection, analysis, and interpretation. The team was linguistically diverse; two were fluent in isiZulu, Sesotho, and English, while one was fluent in isiZulu and English, ensuring participants were interviewed in their preferred language to reduce misinterpretation and increase trust. No prior personal relationships existed between the researchers and participants, and transparency was maintained by clearly outlining the researchers’ roles during interviews. To further strengthen rigour, peer debriefings, collaborative coding, and regular team discussions were conducted to minimise individual bias and to ensure that participants’ voices were prioritised over preconceived interpretations.

### Ethical considerations

Ethical clearance was obtained from the Human Sciences Research Council (reference number: REC 3/22/08/18) and the Johannesburg District Ethics Committee prior to initiating the semi-structured interviews with HCWs. Written informed consent was obtained from participants.

## Results

A total of 23 HCWs participated based on their availability, including eight HIV testing counsellors, two retention counsellors, and five nurses from the facilities. Additionally, eight HIV testing counsellors from the NPOs took part in the study (see Table 1).

**TABLE 1:** Demographics of the healthcare workers (*N* = 23).

Category	Sub-category	*n*	%
Job category	Lay counsellor	18	78.3
	Nurse	5	21.7
Age (years)	20–40	13	56.5
	40+	10	43.4
Setting	Facility	15	65.0
	NPO	8	35.0

NPO, non-profit organisation.

### Perceived barriers and facilitators by healthcare workers in the implementation of index contact testing

**FIGURE 1:**
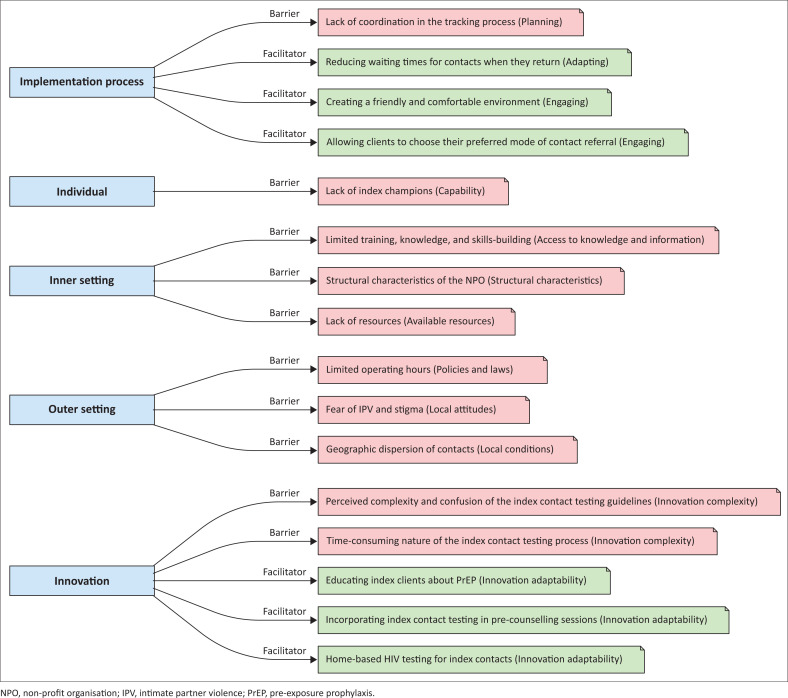
Summary of key barriers and facilitators by domain.

Barriers and facilitators were classified according to the Consolidated Framework for Implementation Research domains (see Figure 1).

#### Innovation domain

**Innovation complexity:** HCWs found the index contact testing process complicated, noting that they only partially followed the guidelines because of confusion about the implementation steps and challenges with documentation. Lay counsellors also highlighted that the time-intensive nature of the index offering process posed significant barriers, as it was difficult to integrate into their daily workflows. The time requirement often forced them to shorten counselling sessions and reduce the time allocated for adherence counselling:

‘I do implement index but am unsure if I am implementing it according to the guidelines.’ (Clinic B, retention counsellor 1)

‘When it comes to index contact and index offering, we get confused as counsellors it was not clear which one are we doing first and which comes second.’ (Clinic C, lay counsellor 2)

**Innovation adaptability:** Participants reported adaptations to the original index contact testing model that facilitated implementation, including educating clients about pre-exposure prophylaxis (PrEP) through counselling sessions when offering index contact testing, integrating index contact testing into pre-test counselling, and offering home-based HIV testing for index contacts. Lay counsellors reported that educating clients on PrEP and viral load (VL) suppression motivated clients to share contact information and, in some cases, brought contacts in for testing. Introducing index contact testing during pre-test counselling allowed counsellors to gather information on contacts early, reducing resistance later. Additionally, home-based HIV testing for index contacts enabled HCWs to reach clients in familiar settings, making them feel more comfortable with the testing process. These adaptations increased client acceptance and engagement, improving the implementation success of index contact testing:

‘If they are negative then we encourage they will have to take PrEP because others are planning to have kids in the future, then we tell them that when one is suppressed it’s not easy to infect other people and we have beads, we show them to the patients and teach them about how VL works in the body.’ (Clinic B, retention counsellor 1)

‘So, I prefer a home address and I find that it works because we do get them in the address that they give us.’ (Clinic C, lay counsellor 3)

#### Outer setting

**Policies and laws:** HCWs perceived facility policies limiting operating hours as a barrier to the implementation of index contact testing, as facilities’ operational hours do not align with patients’ needs. Additionally, school-aged index contacts face challenges accessing the facilities due to school-related commitments:

‘… [*S*]ome will say I am only available on weekends.’ (Clinic C, lay counsellor 1)

‘And then the kids because they will say they are at school.’ (Clinic A, nurse 1)

**Local attitudes:** Participants reported fear of intimate partner violence (IPV) and stigma as barriers to the implementation of index contact testing. HCWs from both facilities and NPOs noted that patients are hesitant to share personal information, such as contact details for their partners and children, because of fears of confidentiality breaches and IPV if their HIV status is disclosed. Stigma from partners and children also deters some index clients from providing details of their contacts. This distrust creates negative perceptions and reduces cooperation with the index contact testing modality:

‘… [*S*]ometimes they will tell us that their partners will beat them up if they give away their numbers. And once they tell us that then we classify that one under declined index because of safety reasons.’ (Clinic C, lay counsellor 3)

**Local conditions:** The geographic dispersion of contacts was perceived as a barrier to the implementation of index contact testing. This barrier complicates implementation, as some contacts live in different provinces or countries, making follow-up and offering HIV testing challenging and diminishing the overall effectiveness of the index contact testing approach. Here is one of the quotes from HCWs:

‘… [*S*]he [*index clients*] will tell us that she doesn’t stay with the kids, and if it’s a man he will say the kids stay with the mother in a different location, and sometimes as an NPO in that spot, the kids are going to school and some they stay in another province, so it ends up being difficult.’ (NPO A, lay counsellor 1)

#### Inner setting

**Access to knowledge and information:** Limited training, knowledge, and skills-building were reported among HCWs in both healthcare facilities and NPOs. Between 2019 and 2021, many lay counsellors received only one round of training with no refresher courses, while at NPOs, some had never received training in index contact testing. In healthcare facilities, initial training was attended only by lay counsellors, with most nurses excluded, leaving them unprepared to support the process. This gap has led to a lack of understanding of index contact testing steps, its purpose, target population, and distinctions between index clients and contacts. Some HCWs were also uncertain of who could be offered index. While retention counsellors were trained more recently in 2023 and 2024, the absence of ongoing, comprehensive training for all HCWs, especially lay counsellors and nurses, creates significant knowledge and skill gaps that hinder effective implementation:

‘I never got trained on how to do index testing properly, what I do when it comes to index is what I have been told by our CLO [*Community Linkage Officer*], so I am not 100 percent confident with what I am doing.’ (Clinic D, nurse 1)

**Structural characteristics:** Structural constraints within their organisations were identified by NPOs as barriers to implementing index contact testing effectively. NPOs do not initiate treatment for clients living with HIV and instead refer them to healthcare facilities, making it challenging to ensure people with positive tests start treatment. Additionally, the mobile nature of NPO staff, who frequently shift locations within the community, makes it difficult for clients to locate them to bring in contacts for testing:

‘It does work, but not as much because when we test a person in a gazebo next week, we are no longer in that place that we were last week. So, I always feel like it’s better if it is in the clinic because most of the time, we are not stationed in one area.’ (NPO A, lay counsellor 1)

‘No, we don’t initiate on site but what we do is that we write them a referral letter and we tell them that they must go to their nearest clinic … And the linkage officer does follow up on the patient as well to see if they have been to their nearest clinic.’ (NPO D, lay counsellor 1)

**Available resources:** HCWs at both the facilities and the NPOs reported a lack of resources for comprehensive implementation as a barrier. They lacked resources such as cell phones and transport to trace patients, and index registers for keeping track of clients and monitoring:

‘… [*A*] lack of logistical capacity to reach more contact cases in communities.’ (NPO A, lay counsellor 2)

‘Maybe, we also need another phone for phoning those patients. It’s just that we, in our department, don’t have another phone. We only have the phone in the office. This means it’s a bit difficult because sometimes you find that they are busy using the phone in the office.’ (Clinic B, nurse 1)

#### Individual domain

**Capability:** The absence of formally assigned index contact testing champions in healthcare facilities and NPOs was reported as a barrier. Without clear designation, there is a gap in leadership and structured support. This lack of clarity on designation and designated expertise leaves HCWs without the guidance they need:

‘I think we all know index testing at the same level. I can say that we don’t have an index testing champion at the moment.’ (NPO C, lay counsellor 1)

‘There is no leader of the index.’ (NPO B, lay counsellor 3)

‘No, we don’t have an index champion.’ (Clinic C, lay counsellor 3)

#### Implementation process

**Planning:** Lay counsellors identified a lack of coordination in tracking index contacts as a barrier. When index contacts return to the facility, they are often tested by a different counsellor, which complicates the tracking, recording, and reporting processes. Suboptimal planning of this process has led to this challenge:

‘I talked to my index and found out they were positive and told them to come back with their partners but when they did, they found someone else.’ (Clinic C, lay counsellor 2)

‘The problem I see is that when the partner comes to the clinic and you told him that when he or she comes, they must ask for a specific counsellor, they come here as someone who is coming to test, and they go to a different counsellor and that person is my contact.’ (Clinic A, lay counsellor 1)

**Adapting:** In this construct, one facilitator was reported as reducing waiting time for clients/contacts when they return. Participants stated that this facilitator makes the process more efficient and less burdensome for patients. This facilitator demonstrated an adaptation specifically designed to improve client experience and streamline the index contact testing process. The lay counsellors mentioned:

‘Yes it has helped a lot and those ones we attend to them quickly when they come with their partners.’ (Clinic C, lay counsellor 4)

‘[*W*]ill take the patient through the process as quickly as possible without having to join the lines then, in that case, self-management works there because the person does not spend so much time in the facility.’ (Clinic A, lay counsellor 1)

**Engaging:** A key facilitator at facilities was the creation of a friendly and comfortable environment for patients, along with allowing clients to choose their preferred method of contact referral. HCWs emphasised that fostering a welcoming and non-judgmental atmosphere encourages index contacts to feel at ease and participate actively in the testing process. This highlights the importance of engaging with patients. The participants highlighted:

‘[*T*]he only thing that you can improve in the index is having to create a friendly and comfortable environment for the patient so that they can be open enough and disclose their partners and children and give the contacts’ (Clinic B, lay counsellor 2)

Moreover, HCWs engaged with patients by asking when they plan to bring their contacts and inquiring about their preferred referral method. HCWs observed that most index clients preferred the client referral method for notifying their contacts. This mode of referral was also considered the most effective in index contact testing. They explained that clients feel more comfortable handling the referral process on their own, as this allows them to disclose their HIV status in a way and at a time that feels right for them. This approach gives clients greater control over the disclosure, helping to reduce stress and maintain privacy, as they can choose how to share sensitive information with their contacts. Many clients also felt more at ease bringing their partners to the facility themselves for HIV testing. Below are participants’ quotes on the preferred mode of contact referral:

‘They choose the first one where they would come with a partner.’ (Clinic A, lay counsellor 1)

‘OK, in most cases, they usually say they will talk to their partners first …’ (Clinic B, lay counsellor 2)

## Discussion

This study reveals the complex interactions among various Consolidated Framework for Implementation Research domains (particularly innovation, outer, and inner settings) and highlights the importance of targeted innovations and supportive implementation strategies to improve testing uptake across different healthcare environments. The innovation, outer setting, and inner setting domains had the most important barriers, while the innovation and implementation process domains presented the most important facilitators.

In the innovation domain, primary barriers included perceived complexity and confusion around index contact testing guidelines, as well as the time-consuming nature of the process, which deterred HCWs from fully implementing index contact testing. Limited time amid heavy workloads has previously been reported as a barrier.^12^ These findings suggest that streamlining guidelines and simplifying procedures could reduce provider burden and improve adoption. Key facilitators included educating index clients on PrEP, incorporating index contact testing into pre-counselling sessions, and offering home-based HIV testing for index contacts: these streamlined multiple counselling processes and improved client engagement.

Barriers in the outer setting included fears of IPV and stigma, as well as challenges posed by the geographic dispersion of contacts, which complicates outreach and testing. Fears related to divorce, societal stigma, long distances, and logistical obstacles to tracing contacts have previously been identified as major barriers.^1,12^ South Africa has a high prevalence of sexual and gender-based violence (SGBV) and HIV. Approximately 14% of individuals experience sexual violence in their lifetime, while 16% endure physical violence, with SGBV being particularly common among those accessing HIV testing and treatment services.^30^ In such settings, implementing index contact testing requires a trauma-informed and client-centred approach that prioritises safety, confidentiality, and survivor-centred services. This includes pre-screening for IPV risk, offering psychosocial support, and providing assistance for contact tracing. Additionally, training healthcare providers, collaborating with communities to reduce stigma, and upholding ethical practices are critical for sensitive and effective implementation. A client-centred approach, which addresses individuals’ unique needs and challenges, can enhance trust, engagement, and the effectiveness of index contact testing.

Within the inner setting, barriers were primarily because of limited training, insufficient knowledge, and inadequate skill-building among HCWs, along with a lack of resources, hindering effective implementation. A lack of adequate training, insufficient elicitation skills, a low number of people trained, and inadequate knowledge have previously been reported.^1,12^ In our study, HCWs reported specific resource shortages, including cell phones for telephonic follow-up, transport for patient tracing, and index registers for documentation. These limitations could hinder effective follow-up with index clients and create gaps in recording index information.

The client referral method, also known as passive referral, wherein the index client brings their contact for testing, emerged as a preferred contact tracing strategy for patients. This approach was favoured by both HCWs and index cases in studies conducted in Malawi and other regions.^1,31^ The client referral method is an appropriate strategy in South Africa, with its high prevalence of SGBV, as it empowers the index client to control the disclosure process. By allowing clients to decide how and when to engage their contacts, this method reduces the risk of coercion or exposure to IPV. However, the focus on this method has anecdotally been associated with low return rates without intensive support.

Index contact testing is influenced by multi-level factors and there is a need to develop strategies targeting each level. To improve index contact testing initiatives, several recommendations are proposed: first, increasing awareness of PrEP among clients and incorporating discussions about index contact testing into pre-counselling sessions will help normalise the process. Expanding access to home-based index contact testing will further improve accessibility for clients. It is also vital to provide comprehensive and regular training for all staff involved in the HIV programme to ensure they are well equipped to support testing efforts. Additionally, securing essential resources, such as cell phones and transport for home-based testing, is crucial for effective implementation. Addressing concerns related to stigma and IPV through targeted outreach will foster a more supportive environment. Finally, creating a welcoming environment within healthcare facilities and reducing waiting times will encourage greater participation. By implementing these strategies, healthcare providers can effectively overcome barriers and enhance the outcomes of index contact testing initiatives.

This study has several limitations. The findings are based on the perspectives of HCWs, which may be subject to response bias and may not fully capture the complexities of the barriers and facilitators experienced in practice. We did not capture the perspectives of all stakeholders involved, including patients and community members, which would provide a more holistic understanding of the testing process. The study’s scope was limited to specific healthcare facilities and NPOs, which may limit the generalisability of the results to other contexts or regions.

## Conclusion

Several barriers, including complicated guidelines, time constraints, stigma, IPV, geographic challenges, and limited training and resources for HCW, hindered the effective implementation of index contact testing in our setting. However, key facilitators such as educating clients about PrEP, integrating index contact testing into pre-counselling sessions, offering home-based testing, and fostering positive provider attitudes can enhance the index contact testing process and improve client engagement. The preferred referral method was client referral, where index clients bring their contacts for testing, which has shown to be a useful and acceptable approach. Policymakers, HCWs, and programme implementers should prioritise the adequate implementation of guidelines and training programmes that address the barriers to index contact testing.
